# A Sustainable Banana Peel Activated Carbon for Removing Pharmaceutical Pollutants from Different Waters: Production, Characterization, and Application †

**DOI:** 10.3390/ma17051032

**Published:** 2024-02-23

**Authors:** Osamah J. Al-sareji, Ruqayah Ali Grmasha, Mónika Meiczinger, Raed A. Al-Juboori, Viola Somogyi, Khalid S. Hashim

**Affiliations:** 1 Sustainability Solutions Research Lab, Faculty of Engineering, University of Pannonia, Egyetem str. 10, H-8200 Veszprém, Hungary; 2 Environmental Research and Studies Center, University of Babylon, Babylon, Al-Hillah 51001, Iraq; 3 Research Group of Limnology, Center for Natural Science, Faculty of Engineering, University of Pannonia, Egyetem u. 10, H-8200 Veszprém, Hungary; 4 NYUAD Water Research Center, New York University Abu Dhabi, Abu Dhabi 129188, United Arab Emirates; 5 Water and Environmental Engineering Research Group, Department of Built Environment, Aalto University, P.O. Box 15200, FI-00076 Espoo, Finland; 6 School of Civil Engineering and Built Environment, Liverpool John Moores University, Liverpool L3 2ET, UK; 7 Department of Environmental Engineering, College of Engineering, University of Babylon, Babylon, Al-Hillah 51001, Iraq; 8 Civil Engineering Department, Dijlah University College, Baghdad 00964, Iraq

**Keywords:** adsorption, reusability, water treatment, organic micropollutants, emerging contaminants, waste

## Abstract

Due to the growing concerns about pharmaceutical contamination and its devastating impact on the economy and the health of humans and the environment, developing efficient approaches for removing such contaminants has become essential. Adsorption is a cost-effective technique for removing pollutants. Thus, in this work, banana peels as agro-industrial waste were utilized for synthesizing activated carbon for removing pharmaceuticals, namely amoxicillin and carbamazepine from different water matrices. The chemically activated carbon by phosphoric acid (H_3_PO_4_) was carbonized at temperatures 350 °C, 450 °C and 550 °C. The material was characterized by several techniques such as scanning electron microscopy with energy dispersive X-ray spectroscopy (SEM-EDS), Fourier transform infrared spectroscopy (FTIR), Boehm titration, point of zero charge (pH_PZC_), BET surface area (S_BET_), the proximate and ultimate analyses, X-ray powder diffraction (XRD), and thermos-gravimetric analysis (TGA). The SEM of banana peel activated carbon (BPAC) depicted a semi-regular and heterogeneous morphology, characterized by an abundance of pores with diverse forms and sizes. Boehm titration revealed an increase in the amounts of acidic groups by 0.711 mmol/g due to activation by H_3_PO_4_. FTIR recorded different peaks suggesting significant modifications in the spectroscopic characteristics of the BPAC surface due to the successful activation and adsorption of the pollutant molecules. The pHpzc of BPAC was calculated to be 5.005. The S_BET_ surface area dramatically increased to 911.59 m^2^/g after the activation. The optimum conditions were 25 °C, a materials dosage of 1.2 g/L, a saturation time of 120 min, a pollutants mixture of 25 mg/L, and a pH of 5. Langmuir exhibits a slightly better fit than Freundlich with a low value of the residual sum of squares (SSE) and the data were better fitted to the pseudo-second-order kinetic. Furthermore, the efficacy of BPAC in eliminating pharmaceuticals from Milli Q water, lake water, and wastewater was successfully investigated over the seven cycles. The results of the present work highlighted a potential usage of agro-industrial waste in eliminating organic micropollutants while exhibiting sustainable management of this waste.

## 1. Introduction

Water is essential for sustainable and socio-economic development since it plays a pivotal role in every dimension of growth in the economy and social well-being. Ensuring the quality of potable and safe water is vital for public health, regardless of where this resource is being utilized [1]. However, manufacturing processes, which play a crucial role in the economy, produce by-products and residues that include compounds that have the potential to cause environmental damage and pollute water bodies if not effectively controlled [2].

Pharmaceuticals have been an influential driver for worldwide scientific and technical progress, leading to longer life spans, the recovery of millions from life-threatening illnesses, and an improvement in overall quality of life [3]. Because of their high visibility, they are currently becoming more prevalent as rapidly spreading environmental contaminants. Pharmaceutical compounds have been detected in almost all biological systems across all continents in the last thirty years [4]. This includes wastewater, groundwater, surface water, and sludge. The problem of pharmaceutical pollution in the ecosystem is complex due to the many sources of pharmaceuticals and the extensive use of therapeutic agents [5]. The total count of pharmaceutically active chemicals was determined to be 11,926, with 713 of these chemicals found in wastewater [6]. Carbamazepine, one of the most well-known antiepileptic compounds, also chemically very stable, has been found in wastewater effluents all over the globe [7]. According to European regulations, it is categorized as a harmful chemical to aquatic organisms [8]. Amoxicillin has been linked to the occurrence of hemorrhagic, sometimes inflammatory colitis, mostly affecting the ascending colon [9].

Various methods were reported for eliminating contaminants from aquatic environments such as ultrafiltration, nanofiltration, reverse osmosis [10], adsorption [11], advanced oxidation [12], and photocatalytic technique [13,14]. Adsorptive treatments are recognized and widely accepted techniques for use in wastewater remediation because of their excellent operating efficiency [15]. Activated carbon is widely recognized as the most effective adsorbent among the options available for commercial use, primarily because of its exceptional ability to eliminate pollutants. Still, the exorbitant manufacturing costs and limited economic viability render it unfeasible for widespread implementation. Utilizing natural substances derived from renewable resources, such as agro-industrial waste that are often underutilized and lack economic value, has shown great potential for the advancement of biosorbents [15,16].

Biosorbents provide several benefits over traditional chemical adsorbents in water treatment. These materials are inexpensive, often capable of decomposing naturally, and readily accessible and produced. A wide variety of biomass, including bacteria, fungus, algae, industrial waste, natural waste, and agricultural waste, could be employed as raw material for the generation of biosorbents [15,17]. These biosorbents possess exceptional capabilities in removing an array of contaminants from wastewater [18].

Banana is a generic name that includes all wild species, cultivars, and landraces that are members of the Musaceae family and the Musa genus. Musa cavendishii, also known as the cavendish banana, Musa paradisiaca, also known as the plantain, and Musa sapientum, sometimes known as the chunkey banana, are the species that are farmed the most all over the globe [19]. The banana is the second most farmed fruit in the globe, with a total production of 124.97 million tons in 2021 [20]. The cultivation of bananas results in the production of a significant quantity of biomass that is not intended for use, including rachis, leaves, pseudostem, and fruit peels. It has been estimated that the bulk of the fruit accounts for just twelve percent of the overall mass of the banana tree [21]. The peels account for between 35 and 50% of the overall weight of the fruit, which is a kind of biomass that is not being used and is often thrown away as waste [22] and was made accessible for the creation of novel biosorbents. Researchers examined the possibility of removing various pollutants from banana residues, including metals, organic micropollutants, and dyes [19].

Banana peel has been widely employed as a potential waste for pollutant removal such as heavy metals, radioactive elements, pharmaceuticals, dyes, crude oil, phenolic compounds, fluoride, and aflatoxins [23]. For instance, the removal of polycyclic aromatic hydrocarbons (PAHs) such as fluorene, naphthalene, and phenanthrene using banana peel activated carbon was studied [24]. All three PAHs reached equilibrium at 60 min. At a concentration of 20 mg/L of PAH solution, fluorene, and phenanthrene were efficiently removed at 0.206 g/L and naphthalene at 0.333 g/L. All three PAHs were adsorbable at low pH because the adsorbent surface remained positively charged, which helped attraction (electrostatic) between the negatively charged PAH adsorbate and the adsorbent. Ingole and colleagues (2017) used activated carbon derived from banana peels (BPAC) to remove phenol [25]. When the starting concentration rose from 50 mg/L to 500 mg/L, BPAC removal and adsorption capacity dropped from 83% and 6.98 mg/g to 60% and 48.58 mg/g. The best phenol removal occurred with the adsorbate solution pH6.0. It was shown that increasing temperature reduced phenol adsorption onto BPAC, indicating spontaneous and exothermic adsorption.

However, most of the published work did not take into account the removal of pharmaceutics mixture (especially amoxicillin and carbamazepine) as widely detected contaminants in the environment. Although selecting two pharmaceuticals might not be a true representation of the reality where many of these contaminants co-exist, the goal of the study is to thoroughly evaluate the feasibility of the developed adsorbent for removing such contaminants in different environmental settings. Additionally, selecting pharmaceuticals that belong to two different classes (i.e., antibiotics and anticonvulsants) helps in having a better evaluation of the adsorbent capacity for different chemical structures and it is a common practice for lab scale studies. For instance, Nielsen et al. examined the activated carbon adsorption capacity of sulfamethoxazole and carbamazepine [26], while Delgado et al. assessed the powdered activated carbon capacity to remove carbamazepine and sildenafil citrate [27]. Most of the studies in the literature focused on one type of water matrix and neglected other types (for instance, lake water). Thus, this study is dedicated to developing an environmentally friendly active carbon from agro-industrial waste (banana peels) and utilizing it to remove complex pollutant mixtures from different wasters. The study first activated the materials with phosphoric acid (H_3_PO_4_) and evaluated the materials with different pyrolysis temperatures. Then, the optimum parameters were tested including materials dosages, pollutant concentration, pH, temperature, and reaction time with respect to the removal efficiency. The isotherms and kinetics were also investigated. Within the optimum conditions, the materials were able to eliminate the target chemicals from water, lake water (Lake Balaton, Hungary), and wastewater for seven cycles.

## 2. Materials and Methods

### 2.1. Materials

The McIlvaine citrate–phosphate solution was prepared using citric acid (C₆H₈O₇) and disodium phosphate (Na_2_HPO_4_) provided by AVANTOR (Radnor, PA, USA). The chemicals obtained from Sigma-Aldrich (Burlington, MA, USA) include sodium hydroxide (≥98%, NaOH), hydrochloric acid (HCl, 37%), sodium bicarbonate (99.5–100.5%, NaHCO_3_), sodium ethoxide (≥95%, NaOC_2_H_5_), and sodium carbonate (≥99.5%, Na_2_CO_3_). Amoxicillin (C_16_H_19_N_3_O_5_S, CAS No.:26787-78-0), phosphoric acid (H_3_PO_4_,85%, CAS No.:7664-38-2), and carbamazepine (C_15_H_12_N_2_O, CAS No.: 298-46-4) were supplied by Sigma-Aldrich. Banana peels (BP) were obtained from local supermarket in Veszprém, Hungary free of charge. The unreported substances employed in this study were acquired from Sigma-Aldrich. Whatman^®^ glass microfiber filters were utilized to separate the adsorbents from the treated solution. The chemical compounds and reagents utilized were of analytical quality and did not need further purification. Throughout the testing procedures, the MilliporeDirect-Q^®^ 5 UV (Merck KGaA, Darmstadt, Germany) purification system was used to provide ultra-pure water (MQ) with a resistivity of at least 18.2 MΩ·cm.

### 2.2. Biomass and Activation

To remove surface dirt and other contaminants, banana peels (BP) were cut into pieces 2 cm in length and washed thoroughly with both tap water and deionized water. Then, the materials were dried for a period of 24 h in an oven at a temperature of 100 °C, and they were ground and sieved in order to obtain particles with diameters that were less than 0.150 mm. Phosphoric acid (H_3_PO_4_) is a widely used activating agent that has been extensively investigated for its role in the production of activated carbons from agricultural waste [28]. The dried BP (25 g) was soaked in 500 mL acid solutions (25% *v*/*v* of acid) in one liter Erlenmeyer flask following the earlier reported method with a slight modification [29]. The experiment was conducted on an electric hotplate for a period of 4 h at a temperature of 80 °C. After that, the impregnation agent was withdrawn, and the impregnated BP was oven-dried at 110 °C for 6 h. Following that, BP was carbonized at temperatures 350 °C, 450 °C, and 550 °C for 30 min. This process was carried out by using a cylindrical stainless steel reactor positioned within a furnace. Afterward, activated carbon derived from the banana peel (BPAC) was washed and rinsed several times via hot distilled water to neutralize the materials to pH (6–7) and to remove residual chemicals. It was then subjected to oven-drying at a temperature of 105 °C to remove any remaining moisture and volatile substances that may have adhered to it [30]. The BPAC was stored in a tight container for further research.

### 2.3. Characterization

The Fourier transform infrared spectroscopy (FTIR) spectra of the BPAC before and after the adsorption process were investigated to evaluate the functional groups present on the BPAC surface. The analysis was performed in a Nicolet™ iS™ 5 FTIR Spectrometer, Thermo Fisher (Waltham, MA, USA), using iD7 ATR, in 400–4000 cm^−1^ as a wavenumber range. Prior to each analysis, a blank background spectrum was acquired in order to establish a reference scale for absorption intensity and to perform blank subtraction. The materials were dried at a temperature range of 50 °C for a period of 2 h to ensure thorough elimination of water. The evaluation of the point of zero charge (pH_PZC_) was performed by combining 0.01 g of the biosorbent under investigation with 10 mL of a 0.1 mol/L NaCl solution. The initial pH of a mixture varied within the range of 2 to 10. The dispersions were agitated for 24 h, and the resulting pH values were determined. Every experiment was conducted three times. The pH_PZC_ value was determined as the abscissa point where the curve of ΔpH vs. starting pH intersected the line representing zero. The structural morphologies of the BPAC were characterized using scanning electron microscopy (SEM) (JEOL JIB-4700F) (GENTLEBEAM™ (GB), Tokyo, Japan) energy dispersive X-ray analyzer (EDS). For the studies, every sample was positioned on a stub and covered with a layer of gold prior to SEM inspection. The examined BPAC was fixed onto a conductive carbon tape and coated with gold/palladium (Au/Pd) film for 10 min to enhance their conductivity and obtain high-quality images. Surface area measurements (S_BET_) were conducted via the Brunauer, Emmett, and Teller (BET) method using the Micromeritics (3Flex) instrument (Unterschleissheim, Germany). The measurements were carried out based on the nitrogen (N_2_) gas adsorption technique in a liquid nitrogen environment at 77 K. The presence of oxygen-containing functional groups on the adsorbent surface (BPAC) was examined employing Boehm titration. The experimental procedures were reported in our work previously [31]. The materials were evaluated using proximate analyses according to the methods specified by the American Society for Testing Materials (ASTM). The ash content was determined using ASTM D3174–04 [32], the moisture content was evaluated using ASTM D3173–03 [33], and the volatile matter was assessed using ASTM D3175–07 [34]. The determination of fixed carbon content included deducting the combined quantities of moisture, ash, as well as volatile matter from a sum of 100%. The adsorbents’ elemental composition was analyzed using an elemental analyzer (Model EA 1108, Thermo Scientific, Waltham, MA, USA) [35]. The quantification was conducted in accordance with the procedure parameters outlined in ASTM D3176 [36]. The X-ray powder diffraction (XRD) was obtained using a D/Max 2500 VB+X X-ray diffractometer (Rigaku, Tokyo, Japan), which was equipped with a Cu (40 kV, 35 mA) source from Rigaku in Tokyo, Japan. The diffraction analyses were performed in the 10–80° (2θ) range. The sample was analyzed utilizing thermos-gravimetric analysis (TA Instrument: TGA 550 Model, New Castle, DE, USA). A quantity of 10 mg of banana peel powder was used, with 10 mL/min of N_2_. The sample was subjected to heating at a rate of 10/min, starting at a temperature of 50 °C and reaching a final temperature of 700 °C.

### 2.4. Adsorption Experiments, Kinetic, Equilibrium Studies and Thermodynamics

In order to develop the most effective method of preparing the banana peel for treatment, adsorption tests were carried out in 0.250 L conical flask at ambient temperature (25 ± 2 °C). The trials involved using 0.18 g of biosorbents, and 150 mL of a solution containing pollutant mixtures at an initial pollutant concentration of 25 mg/L, with the pH adjusted to 5. The contact time was set at 120 min, and the agitation rate was maintained at 150 rpm. To assess the impact of the initial pH on the adsorption process, the aforementioned conditions were used using a pollutant mix solution at various beginning pH values (pH 2–10). These pH values were modified using 0.1 mol/L hydrochloric acid (HCl) or 0.1 mol/L sodium hydroxide (NaOH) solutions. The biosorbent was chosen for the next adsorption studies. The impact of the biosorbent dosage was assessed by utilizing doses of 0.3, 0.6, 0.9, 1.2, and 1.5 g/L, respectively, in a 150 mL solution containing pollutant mixes with an initial concentration of 25 mg/L. The contact period was set at 120 min, and the agitation rate was maintained at 150 rpm. Within the same procedure, further parameters such as temperatures (5, 15, 25, 30, and 35 °C) and pollutants mixture (10, 15, 20, 25, 30, and 35 mg/L) were also evaluated. For pollutant quantification, mixtures were centrifuged, and an aliquot of 2 mL of the supernatant was collected and measured using high-performance liquid chromatography (HPLC) with the setting mentioned earlier in [37]. To calculate the amount of pollutants adsorbed (q_e_), in mg/g, Equation (1) was utilized. Equation (2) represents the percentage of pollutant removal.

(1) $$q_{e}=\frac{V\left(C_{0}-C_{e}\right)}{m}$$

(2) $$Removal\;efficiency\;(R\%)=\frac{100\times \left(C_{0}-C_{e}\right)}{C_{0}}$$

where C_0_ is the initial pollutant concentration in the solution, in mg/L, C_e_ is the pollutant concentration at the equilibrium, in mg/L, m is the mass of BPAC used, in g, and V is the solution volume, in L. Experiments were carried out in triplicate, and the coefficient of variance for the tests was equal to or lower than 2.64%.

The optimum adsorption parameters from the above experiment were used to eliminate a mixture of pharmaceuticals in real samples except for the pH, which was left without making any adjustments. The full characterization of the tested different water matrices was reported in our previous work [38]. Then, an amount of the mixture (1 mL) was collected, subjected to centrifugation, and then evaluated using HPLC technique. The investigations were run three times, and the standard deviation was less than 2.08%.

The kinetic studies involved incorporating 0.18 g of absorbent with a pollutant solution that had a concentration of 25 mg/L and a pH value of 5. The mixtures were placed under an agitation rate of 150 rpm and left at ambient temperature (25 ± 2 °C) for a set duration (ranging from 10 min to 120 min). Following each occurrence, the liquid portion (1 mL) was regularly collected and examined using HPLC. The isotherms, pseudo-first- and second-order, and the thermodynamic were assessed using equations in Table 1. In addition, experiments were conducted to determine the biosorption isotherms. A 0.18 g amount of the biosorbents was combined with pollutant solutions ranging in starting concentration from 5.0 to 120 mg/L. The mixture was shaken for 12 h at a temperature of 25 ± 2 °C at 150 rpm (pH5. A variation coefficient of less than 6.04% was found among the runs, which were carried out in triplicate.

The thermodynamic experiment also included conducting biosorption tests at various temperatures (10 ± 1, 20 ± 1, 30 ± 1, and 40 ± 1 °C). Each test was carried out in a 100 mL glass vial containing 0.05 g of adsorbent material and 40 mL of a 25 mg/L solution of pollutants in water. The solutions were agitated in a shaker incubator at a speed of 150 rpm for 24 h at the specified temperature. Subsequently, each combination was subjected to centrifugation, and 1 mL of the solution that formed above the adsorbent was obtained and examined using HPLC. The tests were run in triplicates. The Gibbs free energy change (ΔG°), change in enthalpy (ΔH°), and change in entropy (ΔS°) during biosorption were determined using Equations in Table 1.

### 2.5. Desorption Experiments

The desorption procedure was implemented to assess the reusability of polluted banana peel after batch adsorption tests. The batch desorption tests were performed in 100 mL Erlenmeyer flasks that had been cleaned and dried. Water with a pH of 7 and ethanol as solvents were used. The specified quantity of BPAC was placed into 25 mL of solvent and agitated at a speed of 150 rpm for 6 h. The drying of the samples was at 105 °C for one day. During the runs, the flasks were sealed with parafilm to prevent the solvent from evaporating. Control experiments, conducted without the inclusion of activated carbon, were carried out to evaluate the potential influence of adsorption on the glass surfaces. The desorption % was determined using the following equation.

$${Desorption(\%)}_{BP}=\frac{100\left(C_{des}\right)}{C_{sorb}}$$

where desorption (%), BP is the desorption yield (%), and C_des_ and C_sorb_ are the desorbed BP concentration and sorbed pollutants concentration (mg/L).

## 3. Results and Discussion

### 3.1. Characterization

**Figure 1:**
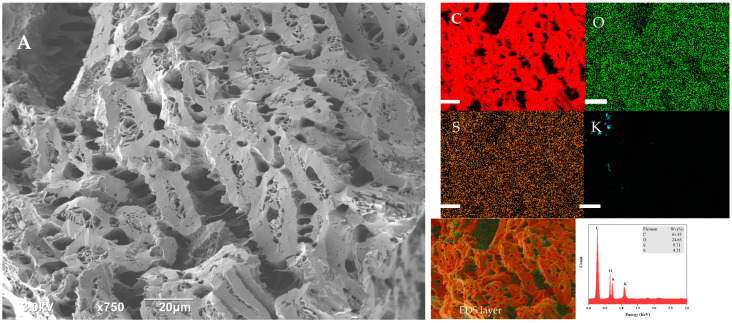

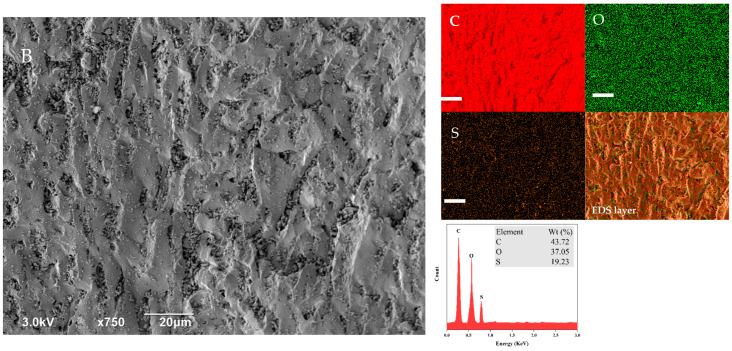
SEM-EDS for BPAC before the adsorption (**A**) and BPAC after the adsorption. (**B**) The EDS scale is 20 µm.

The SEM analyzed the morphological composition of activated carbon derived from bananas (Figure 1). The SEM picture depicted the activated carbon outside as having a semi-regular and heterogeneous morphology, characterized by an abundance of pores with diverse forms and sizes. On the other side, it can be seen that the SEM picture after the adsorption was full in some pores and still there were some available sites. This could also indicate that there is still some location that could be available for chemical adsorption. The EDS is an analytical method used for elemental composition analysis and chemical characterization of activated carbon indicating the presence of carbon, oxygen, silicon, and potassium in banana peels derived activated carbon (Figure 1). The study found comparable outcomes reported by Hussain and colleagues (2023). The study also reported that the silicon content in BPAC corresponds to the silicon content present in the original fruit peels [46]. It is also noticed that the content of S has increased after the adsorption process, which is indicative that a chemical reaction has occurred. The appearance and disappearance of elements or increase and decrease in their count on the EDS spectrum after adsorption is a common observation in inorganic [47,48] and organic [49] adsorption studies. It indicates their involvement in the interaction between adsorbate and adsorbents.

**Figure 2:**
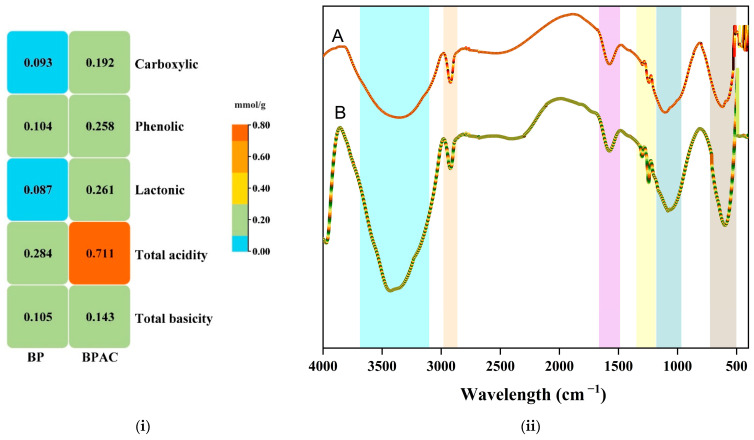
Boehm titration for BP and BPAC (**i**) and FTIR for BPAC before the adsorption (A) and BPAC after the adsorption (B) (**ii**).

Activated carbons are often characterized by the presence of oxygen-containing functional groups, which have a significant impact on the surface characteristics and adsorption capabilities of the carbons. Boehm’s titration for BPAC determined that the acid and basic groups present were carboxylic, lactonic, and phenolic at 0.192, 0.261, and 0.258 mmol/g, respectively. The overall concentration of acidic groups of BPAC was 0.711 mmol/g, whereas the concentration of total basicity was 0.143 mmol/g. The data directly demonstrate that the BPAC surface has a greater abundance of acidic groups compared to basic groups. Furthermore, it has been shown from Figure 2i that the total acidic groups of BPAC have elevated by 2.5 times compared to BP, and by 1.36 times in terms of total basicity. The surface is thus predominantly acidic as suggested by the pH_pzc_ (5) value obtained. Other studies, for example, Agboola and Bello (2020), worked on banana stalk activation by phosphoric acid to remove ciprofloxacin from aqueous media [50]. The study revealed the total acidity to be 0.699 mmol/g and the total basicity to be 0.158 mmol/g.

An FTIR analysis was performed on the surface characteristics of the BPAC that had been produced. In simple terms, this demonstrated the spectroscopic features of the sample, which included prominent peaks that were attributed to functional groups that affect the adsorption process. The FTIR spectra of BPAC revealed the presence of distinct bands at 3422 cm^−1^ (bonded O–H group), 1575 cm^−1^ (carbonyl group), and 2919 cm^−1^ (secondary amine group), which are related to unique functional groups that likely facilitated the effective absorption of contaminants (Figure 2ii). Prior research has also shown the importance of these functional groups in the adsorption processes [50,51]. Additional bands detected on the BPAC that potentially contributed to the interactions include 1301 cm^−1^ (symmetric bending of CH_3_), 1080 cm^−1^ (stretching of ethers—C=O=C), 1244 cm^−1^ (stretching of –SO_3_), and 622 cm^−1^ (stretching of –CN). An observable alteration in the adsorption band, particularly at 3422 cm^−1^, appeared, suggesting significant modifications in the spectroscopic characteristics of the BPAC surface due to the successful adsorption of the pollutant molecules, which is also observed in other reported work [50]. The FTIR spectra for BPAC before and after adsorption clearly show that the surface interacted with the pollutant molecules corresponding with wider bands observed after adsorption. The same trends have been observed in earlier work [50,52].

The measured BET surface area of raw BP is 0.650 m^2^/g. The measured BET surface area is consistent with the findings of earlier investigations, which found values of 0.7963 m^2^/g [53] and 1.73 m^2^/g [54]. The minimal surface area may be attributed to the challenges associated with degassing the lignocellulosic materials. Measuring the BET surface area of lignocellulosic waste presents challenges due to the early combustion of the powder before reaching the degassing temperature. Consequently, the temperature at which degassing occurs (100 °C) is decreased, leading to a decrease in surface area due to the moisture contents [55]. The limited surface area of BP is a distinctive property of carbonaceous materials [56]. Table S1 shows the S_BET_ for the raw BP and different pyrolysis temperatures. On the other side, and after the carbonization process with three different temperatures, it was observed that a carbonization temperature of 450 °C had the highest BET of 911.59 m^2^/g with better-developed pores. Therefore, the present work was processed with this material for further investigations. Bakar and the co-worker (2021) developed an activated carbon derived from banana peels with different pyrolysis temperatures [29]. The study found that the highest BET surface area was at 470 °C (684 m^2^/g) and 355 m^2^/g, 587, and 503 m^2^/g for 450 °C, 490 °C and 510 °C, respectively. At 450 °C, the use of phosphoric acid in the activation would have evaporated as it exceeds its boiling point of 407 °C [57] so there is no risk of leaching of the remnant acid to the water.

**Figure 3:**
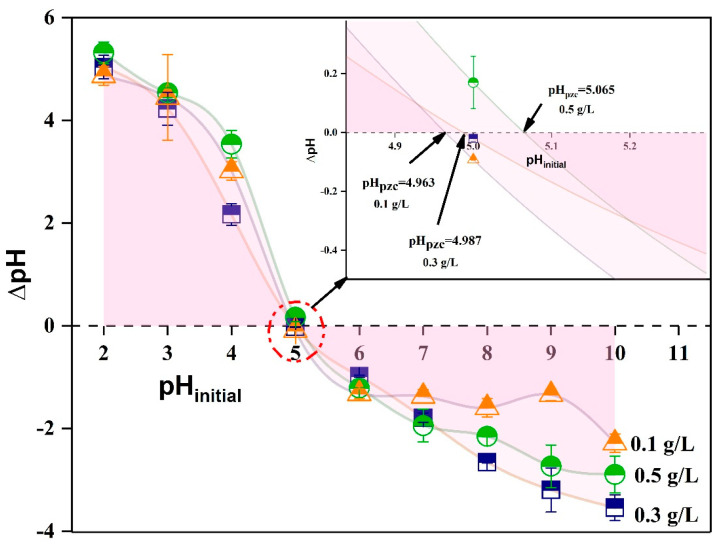
BPAC point of zero charge (pH_pzc_).

The pH at the point of zero charge (pH_pzc_) of BPAC was calculated to be 5.005, based on the average value displayed in Figure 3. These findings suggest that the BPAC surface exhibits a positive charge in the solution until pH 5, after which it gains a negative charge. The pH_pzc_ had an impact on the absorption of pollutant molecules in the experiment [58]. Pathak and Mandavgane (2015) synthesized raw banana peel to remove citric acid from an aqueous solution [55]. The study found that pH_pzc_ was 4.98, which is in line with the present results.

**Figure 4:**
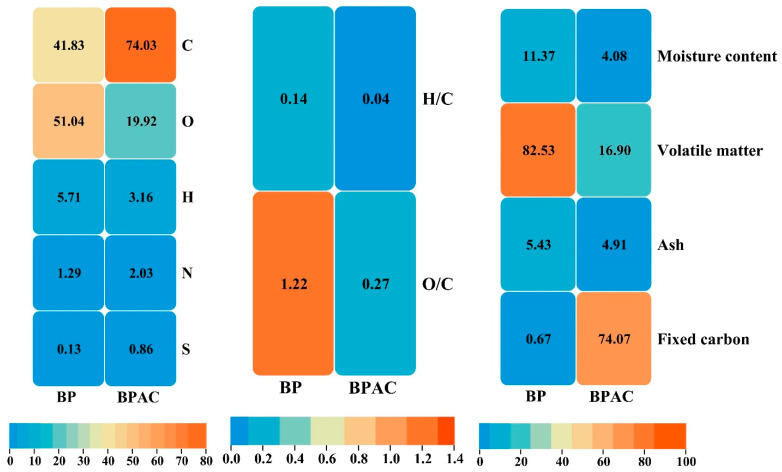
Proximate and ultimate analysis for BP and BPAC.

The examination of the adsorbent’s composition and characteristics is provided in Figure 4, including both the proximate and ultimate analysis. The fixed carbon content increased significantly from 0.67% to 74.07% after the treatment of BP. In contrast, the volatile matter and moisture content dropped considerably from 82.53% and 11.37% to 16.90% and 4.08% for BP and BPAC, respectively. Following treatment, the carbon content experienced a significant rise from 41.83% to 74.03%. However, there was a drop in the content of H, from 5.71% to 3.16%, and O, from 51.04% to 19.92%. These changes suggest an elevated level of carbonization in the materials. The molar hydrogen-to-carbon (H/C) ratio indicates the level of carbonization since hydrogen is mostly linked to plant-derived organic material. The decrease in the H/C ratio after the treatment indicates that the sample experienced carbonization to a certain degree, in comparison to BPAC (H/C = 0.042). The polar groups present on the carbon surfaces serve as sites for water adsorption and facilitate the building of water clusters on the carbon surfaces. Hence, the molar O/C ratio of a char sample could be used as an indicator of its surface hydrophilicity. The O/C BP ratio of 1.220 suggests that the surfaces are more hydrophilic compared to BPAC, which has an O/C ratio of 0.269. The adsorbents with a much higher O/C ratio exhibit a greater concentration of polar groups, which is likely attributed to the presence of carbohydrates. Proximate and ultimate analysis findings were in agreement with other reported work by Pathak and Mandavgane (2015) [55] and Selvarajoo and colleagues, 2020 [59].

**Figure 5:**
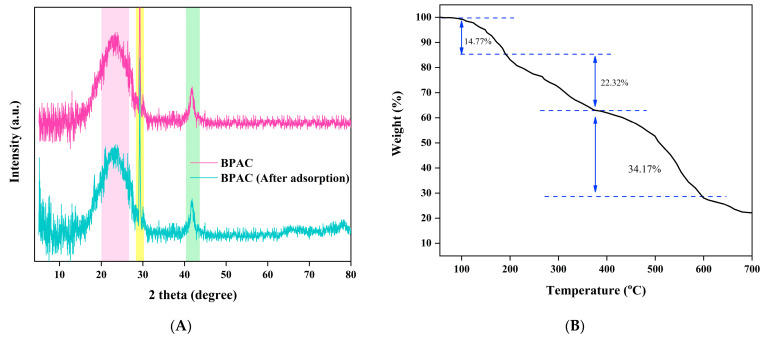
X-ray powder diffraction (XRD) for BPAC before and after the adsorption (**A**) and thermos-gravimetric analysis (TGA) for banana peel (**B**).

Figure 5A shows the XRD spectrum of BPAC and BPAC after the adsorption. It exhibits a large peak at a 2θ of 23.84° (pink). This peak corresponds to the d-spacings in the aromatic area, indicating the presence of a built graphitic structure in BPAC [60]. This peak refers to the (002) graphitic basal plane reflecting graphitic-like microcrystals in BPAC [61]. The presence of this wide peak also results in significant scattering at small angles, which suggests that the high porosity BPAC has amorphous characteristics [62]. The presence of a smaller peak at an angle of 41.86° (green), which corresponds to the (100) plane, suggests the existence of small domains with well-organized graphene sheets [61]. The presence of calcite in the materials is indicated by a sharp peak at 2θ of 29.22° (yellow) [63]. The pharmaceutical adsorption did not cause a tangible change in the XRD spectrum. Similar findings were reported in a prior study conducted by Patel and colleagues, which focused on the use of BPAC for the elimination of ciprofloxacin and acetaminophen [63].

The thermogravimetric (TG) analysis of banana peel is shown in Figure 5B. The first phase of the TG curve was from 50 to 190 °C, resulting in a weight reduction of about 14.77%. The primary cause of this drop is likely water loss and the presence of volatile organic substances, including oils, terpenes, and pigments [64]. The significant weight reduction could additionally be due to the degradation of organic materials such as cellulose and hemicellulose [65]. Following that, the second primary phase of deterioration was seen to take place within the temperature range of 190 to 375 °C. An approximately similar range was reported by Bakar and co-workers [29], who studied the characterization of banana peel activated carbon by phosphoric acid where the temperature range was between 180 and 350 °C. A temperature range of 375–595 °C was attained during the latter phase of conversion. Guimaraes and colleagues [66] stated that banana, sugarcane bagasse, and sponge gourd fibers saw significant mass reduction at temperatures of 300 °C and above. This was attributed to the degradation of hemicellulose and cellulose inside the fibers. Furthermore, the fibers’ deterioration was seen to take place at temperatures above 400 °C, as a result of the breaking of the bonds within the fibers’ lignin.

### 3.2. Influence of Adsorption Parameters

**Figure 6:**
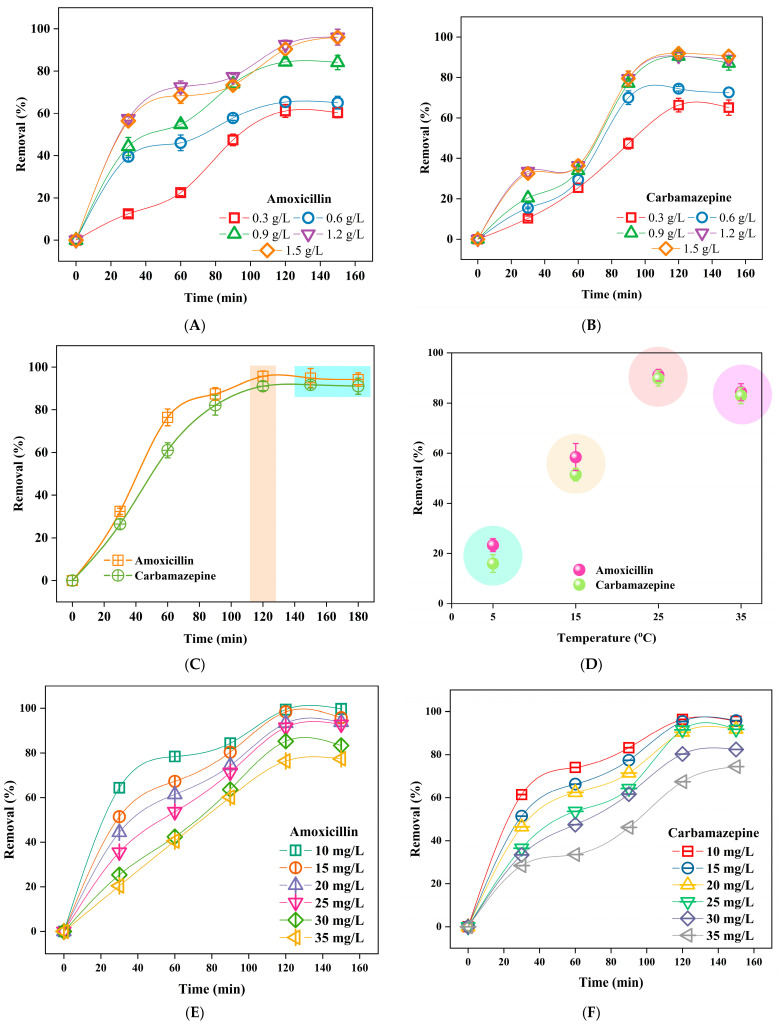

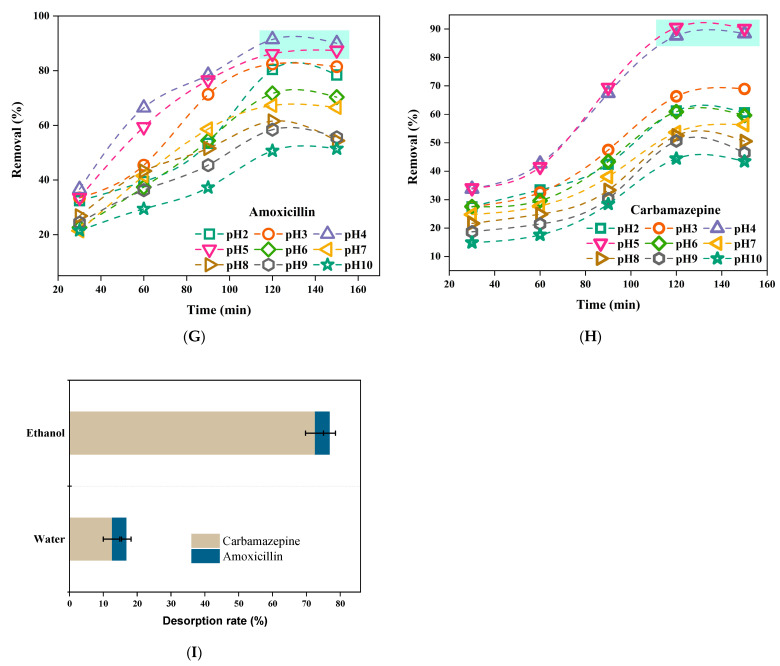
Effect of BPAC dosage on the removal efficiency of amoxicillin (**A**), and carbamazepine (**B**), impact of reaction time on the pollutants removal efficiency (**C**), effect of the temperature on the pollutants removal efficiency (**D**), effect of pollutants initial concentration of the removal efficiency for amoxicillin (**E**) and carbamazepine (**F**), effect of different pH ranges on the removal efficiency for amoxicillin (**G**) and carbamazepine (**H**), and desorption rate for both pollutants (**I**).

The effect of different adsorption parameters on the removal efficiency of amoxicillin and carbamazepine including BPAC dosages, reaction time, temperature, pollutants initial concentration, and different pH ranges illustrated in Figure 6A–H. One of the key components of developing a successful adsorption process is optimizing the quantity of BPAC dosage. The equilibrium connection between adsorbent and adsorbate, and the treatment cost of an adsorbent, could possibly be better predicted with its help. The influence of BPAC dosage on the adsorption of amoxicillin and carbamazepine was examined by introducing different doses ranging from 0.3 to 1.5 g/L (Figure 6). Upon testing the concentrations of 0.3 and 0.6 g/L, both pollutants demonstrated only a slight improvement in removal effectiveness when compared to the other doses (0.9, 1.2, and 1.5 g/L). This is because using smaller quantities of BPAC results in a rapid saturation with contaminants. Increasing the BPAC dosage from 0.9 g/L to 1.5 g/L led to an increase in the effectiveness of amoxicillin elimination. The removal efficiency increased from 84.07% to 96.02% correspondingly. Carbamazepine exhibited a consistent pattern, with its effectiveness increasing from 87.04% to 90.62% at concentrations of 0.9 and 1.5 BPAC g/L, respectively. According to Shao and colleagues (2024), increasing BPAC dosage resulted in a larger surface area for adsorption, which leads to better effectiveness in removing substances [67]. Comparing the two dosages of 1.2 and 1.5 g/L for both contaminants, it is evident that there were only marginal improvements in terms of removal efficiency when the dose of BPAC crossed 1.2 g/L. This indicates that 1.2 g/L is the most effective dosage for removing amoxicillin and carbamazepine using BPAC. Fernandez and colleagues (2015) produced hydrochars from orange peels and used them as bioadsorbents to eliminate diclofenac sodium, salicylic acid, and flurbiprofen [68]. The highest adsorption capabilities for salicylic acid, flurbiprofen, and diclofenac sodium were 0.092, 0.093, and 0.018 mmol/g, respectively, when the optimum adsorbent dose was 0.5 g/L.

The adsorption capacity of an adsorbent is enhanced by extending the contact/interaction period. When the duration of contact expands, the absorption of pollutants rises and eventually reaches an optimal amount [69]. During the adsorption process, there is initially a high level of sorption due to the large number of available adsorbent sites. However, as time continues, the rate of adsorption decreases, resulting in a linear relationship between the amount of adsorption and time. This indicates that the adsorbent has reached its saturation level [70]. Choosing the optimal timing for the adsorption method is crucial as it results in time and cost savings. The removal of both amoxicillin and carbamazepine reached its saturation level at 120 min, and after that, there was no observed increase in the removal efficiency. Thus, 120 min was chosen as an optimal time to process with other parameters. Hodúr and colleagues (2020) studied the reduction in ammonium nitrogen from milking parlor effluent utilizing pomegranate peel powder and achieved a 71% removal rate in about 5 min [71]. Despite this, after 120 min, the system achieved a state of equilibrium, resulting in a maximum elimination of 81.8% of ammonium nitrogen.

An important determinant of whether an adsorption process is endothermic or exothermic is temperature [72]. Adsorption techniques, as a general rule, exhibit endothermic behavior [72]. The sorption capacity of the bio-adsorbent increases as the temperature rises. The increase in sorption capacity could be attributed to the expansion of active sites on the adsorbent’s surface or the movement of pollutant molecules [72]. Increased temperature causes the internal structure of the sorbent to expand, enabling pollutant molecules to penetrate further [73]. A similar tendency was seen in the investigation conducted by Sathishkumar and the team (2007) regarding the elimination of dichlorophenol using palm pith carbon [74]. An important parameter that determines the adsorption capacity is the initial concentration of the contaminants. For amoxicillin, raising its concentration from 10 mg/L to 25 mg/L led to a decrease in the removal efficiency from 99.43 to 91.54%, respectively, and then decreased further. This means that introducing higher concentration leads to quickly occupying the vacant sites on BPAC. The same trend was observed for carbamazepine. Therefore, it was decided to select 25 mg/L for both pollutants as an optimal pollutant concentration. Raising the pH from 2 to 4 results in increasing the amoxicillin removal efficiency from 80.43% to 91.42%. A further increase in pH (pH5) led to a 2% decrease in removal efficiency. Afterward, there was a dramatic drop from pH6 to pH10 to reach 50.65% at pH10. In terms of carbamazepine, there was an increase in removal efficiency from 61.22 to 87.63% when the pH was 2–4. In contrast to amoxicillin, carbamazepine showed a slightly better performance at pH5 to reach a removal efficiency of 90.54%. Comparing the two pollutants’ results with a slight improvement in removal efficiency for pH4 and pH5, a pH5 was selected for other experiments. Ncibi and Sillanpää demonstrated that the CBZ adsorption capacity on mesoporous activated carbon elevated within the pH range of 2–8, rising from 113 mg/g to 188 mg/g [75]. However, when the pH was raised from 8 to 10, the adsorption capacity was reduced to around 140 mg/g [75]. This decrease could be attributed to a mixture of processes, including hydrophobic and π–π interactions between the benzene ring of CBZ and the activated carbon. Naghdi and colleagues reported that there was an enhancement in the elimination of CBZ when the pH level increased from 3 to 9 [76]. The study suggests that H^+^ cations in the medium could be responsible for this increase. Furthermore, at a lower pH, there is an increased concentration of H^+^ ions, which allows for easier interaction between the functional group of CBZ and the H^+^ ions in the medium. Conversely, at a higher pH when H^+^ ions concentration is lower, the hydrogen bonding donor groups on CBZ can interact with hydrogen bonding acceptors or donors in the adsorbent material, thus improving the capacity for removal [75,76]. In this work, the decrease noticed in removal efficiency after pH5 could be attributed to the combination of hydrophobic and π–π interactions between the benzene ring of CBZ and the BPAC. It was stated that the adsorption of amoxicillin onto various adsorbents was enhanced when the pH of the solution rose. This phenomenon is attributed to the change in charge of amoxicillin, transitioning from positive to zwitterionic form [77]. The primary mechanism of adsorption is presumably the electrostatic attraction between the amoxicillin molecules and the surface of the adsorbents. Amoxicillin’s carboxylic groups eventually dissociate at high pH levels, resulting in the formation of a negative charge. The adsorption capacity stays relatively constant as the solution pH increases, starting from a pH of 6 or higher [78]. This phenomenon has been ascribed to the presence of two negative charges on amoxicillin at higher pH levels, which leads to repulsion forces and a decrease in amoxicillin adsorption. The elimination of amoxicillin is more effective at pH levels below the pH_pzc_ (5.005), where there is a prevalence of (amoxicillin±) species and the surface charge is negative. When the pH is higher than the pH_pzc_, the removal of amoxicillin decreases. This could be attributed to the reason that pharmaceuticals and BPAC have negative charges, which results in electrostatic repulsion [79]. Moussavi et al. [80] found that the adsorption of amoxicillin onto NH4 Cl activated carbon (pH_pzc_ = 6.6) reduces as the pH increases. Qin and co-workers (2018) investigated the adsorption characteristics of nitric acid (HNO_3_)-treated coconut shell pellet activated carbon for eliminating Cu(II) and tetracycline [81]. In the acidic pH range of 3 to 6, both Cu(II) (50 mg/L) and tetracycline (250 mg/L) exhibited better adsorption ability near pH6.

The desorption rates for both pollutants were 76.83% and 72.41% for amoxicillin and carbamazepine, respectively (Figure 6I). However, using only water showed a low rate of desorption. Chakraborty et al. [82] confirmed ibuprofen desorption from activated biochar derived from sugarcane bagasse. The desorption process included the use of methanol with continuous agitation at a speed of 130 rpm for a duration of 24 h at a temperature of 25 °C. It remained efficient through four cycles, with rates exceeding 65%. Utilizing a number of different desorbing agents, an investigation was conducted to determine how effective it was in removing contaminants from olive stone waste activated carbon [43]. It was shown that ethanol had the greatest efficiency among the testing agents that were examined, with recoveries of 87.83% for diclofenac and 86.41% for ciprofloxacin, respectively [43].

### 3.3. Adsorbent Reusability with Real Samples Application

**Figure 7:**
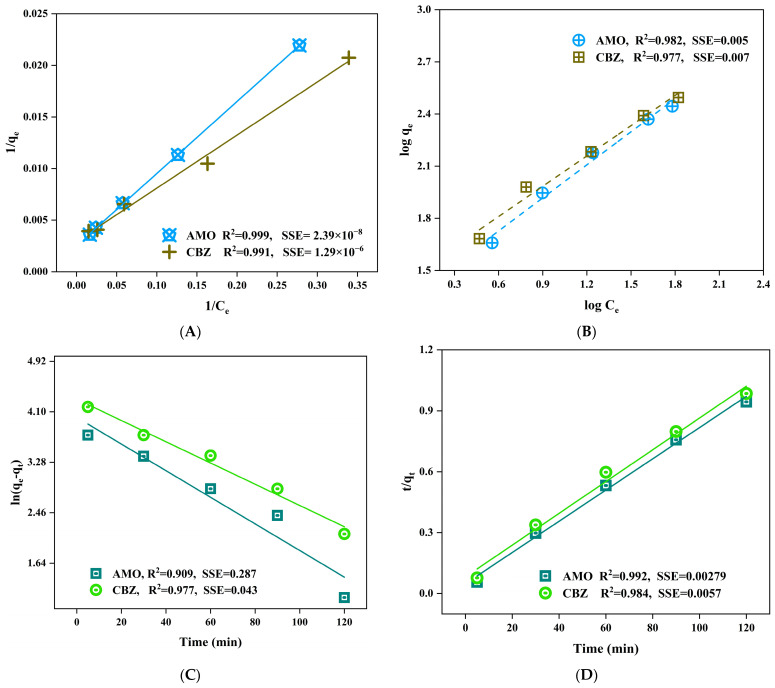

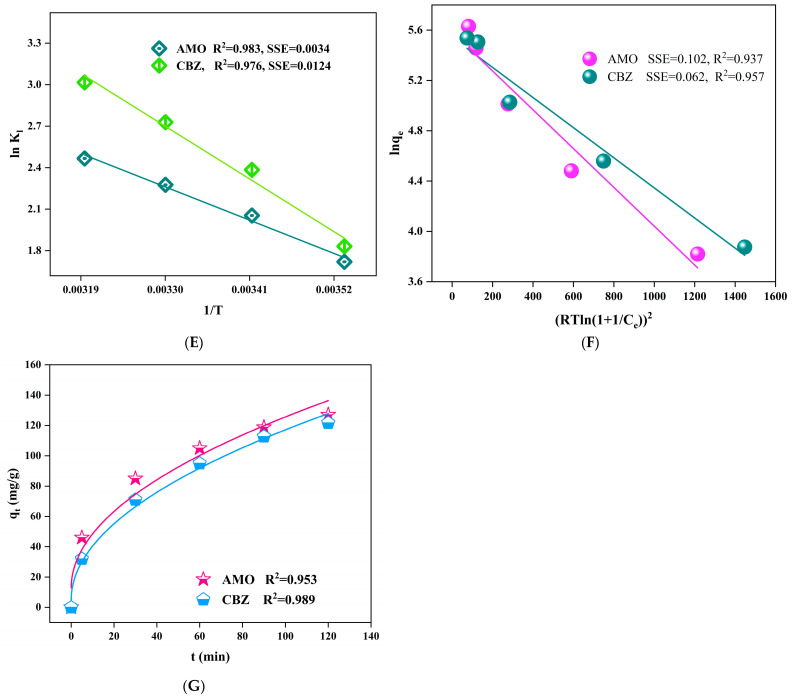
Langmuir (**A**) and Freundlich (**B**) isotherms, pseudo-second-order kinetic (**C**) and pseudo-first-order (**D**) models, thermodynamic (**E**), Dubinin–Radushkevich (D–R) (**F**) for both pollutants, and intra-particle diffusion model (**G**).

Figure 7 illustrates the findings of the fitting experimental data against Langmuir and Freundlich isotherm models. While both isotherm models provide a reasonable explanation for the adsorption data, the Langmuir model is somewhat more accurate. The data were evaluated by the residual sum of squares (SSE), which quantifies the amount of variability in the error, or residuals, of a regression model. A model with a small value indicates a higher level of fit between your model and the data, whereas a larger SSE indicates a lower level of fit between your model and the data. The Langmuir model showed a lower level of SSE with a value of 2.39 × 10^−8^ and 1.29 × 10^−6^ for amoxicillin, and carbamazepine, respectively. Thus, it confirmed that the data were better fitted to the Langmuir model. This suggested the formation of a uniform monolayer of both pollutants onto activated BPAC. The maximal sorption capacity (qm) for amoxicillin was 393.70 mg/g, whereas carbamazepine was 338.98 mg/g. The dimensionless separation factor (RL) establishes the kind of isotherm, which could be classified as irreversible (RL = 0), linear (RL = 1), favorable (0 < RL > 1), or unfavorable (RL > 1) [83]. Table S2 shows the Langmuir, Freundlich, and pseudo-first- and second-order model parameters. According to the results shown in Table S2, the RL calculated value for both pharmaceutical substances was found to be between 0 and 1, which indicates that the adsorption process was favorable. The correlation coefficient for the pseudo-second-order kinetic model was 0.992 and 0.984 for amoxicillin, and carbamazepine, respectively, compared to 0.909 and 0.977 for the pseudo-first-order model. This suggests that chemisorption was the rate-regulating step in the adsorption process. Figure 7 also shows a plot of lnK_1_ versus 1/T for the estimation of the thermodynamic parameters for the adsorption of amoxicillin and carbamazepine. The thermodynamic characteristics responsible for the adsorption of amoxicillin and carbamazepine are shown in Table S3. These parameters include the change in free energy (ΔG°), the change in enthalpy (ΔH°), and the change in entropy (ΔS°) at different temperatures, namely 288, 298, 308, and 318 K. The decrease in ΔG° values that occurred as the temperature climbed is evidence of the favorable influence that temperature has on the efficiency of the adsorption process. The adsorption of amoxicillin and carbamazepine onto BPAC is shown to occur spontaneously, as evidenced by the negative values of ΔG° that were obtained at four different temperatures.

The Dubinin–Radushkevich (D–R) adsorption isotherm model is employed to describe the adsorption phenomenon on the surface that is heterogeneous (Figure 7F), assuming that the energy distribution adheres to a Gaussian distribution. The isotherm model is often used to differentiate between chemical and physical adsorption [44]. The D–R model is an empirical equation that characterizes adsorption by considering the pore-filling mechanism, hence differentiating it from the Langmuir and Freundlich models. The concept is predicated on the premise that it has several layers and incorporates van der Waals forces. The adsorption process could use the apparent energy of adsorption, represented by E (J/mol), to give about changes in the chemical or physical state. As per the D–R isotherm model, physical adsorption takes place when the energy (E) is below 8 kJ/mol. Conversely, chemical adsorption occurs when the energy exceeds 8 kJ/mol. Since both pollutants showed an E value above 8 kJ/mol, chemical adsorption of these pollutants has taken place on BPAC. The diffusion mechanisms of pollutants were identified by fitting experimental data against the intra-particle diffusion model. The intra-particle diffusion state is to be the controlling diffusion mechanism if the plot of qt vs. t is a line passing through the origin (I ≈ 0) [45]. It can be concluded from Figure 7G that intra-particle diffusion is a control mechanism for the tested pollutants. The mixture of processes, including hydrophobic, π–π interactions, and intra-particle diffusion are a control mechanism for the studied contaminants.

### 3.4. Adsorbent Reusability with Real Samples Application

**Figure 8:**
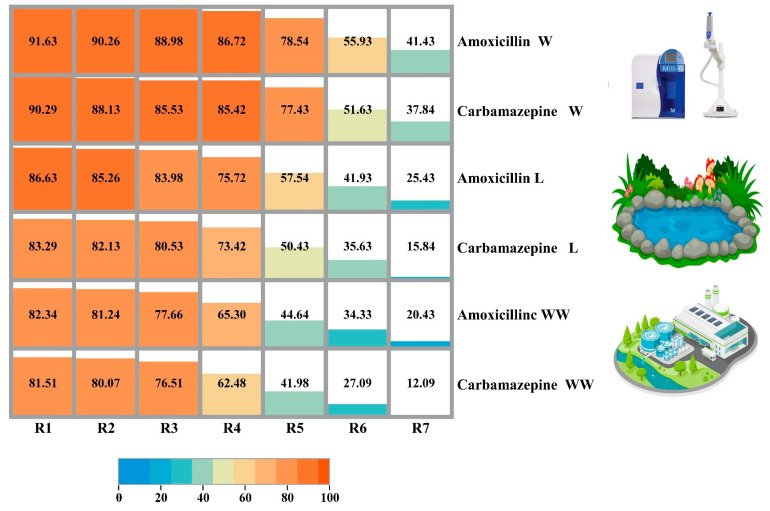
The removal percentage for amoxicillin and carbamazepine using MQ water, lake water (Lake Balaton, Hungary), and wastewater. The SD was less than 3.18% within all cycles. W refers to water, L is lake water, and WW is wastewater.

When an absorbent is reused, it not only helps the sustainability of the process economically, but it also allows for the potential of the adsorbent to be exploited in an appropriate manner [84]. In particular, the fact that bio-adsorbents derived from fruit could be reused not only helps to enhance the cycle process but also serves to minimize the cost of treating water. It is necessary to perform the re-adsorption and desorption procedures several times in order to reuse the bio-adsorbent successfully. Amoxicillin and carbamazepine were tested in three different water matrices namely: MQ water, lake water, and wastewater. Figure 8 illustrates the removal percentage for amoxicillin, and carbamazepine using MQ water, lake water, and wastewater within different cycles. Further water characteristics can be found in [38]. A total of seven successful cycles were tested in terms of pollutant removal for the three water matrices. During the first cycle, more than 90% of the pollutant mixture was obtained when MQ water was used. However, the removal percentage was around 6% less in lake water and 9% in the case of wastewater. The average removal percentage of both pollutants was more than 86.06% in the case of MQ water during the fourth cycle while it was 74.57% and 63.89% for lake water and wastewater, respectively. Following these cycles, there was a dramatic decrease in terms of the removal efficiency of both pollutants for the lake water and wastewater reaching 53.98% and 43.31%, respectively. On the other side and within the same cycle, the pollutant removal in terms of MQ water was approximately constant. The last two cycles (sixth and seventh) showed a decrease in terms of removal efficiency for amoxicillin and carbamazepine to reach seventh cycles around 41.43% and 37.84%, 25.43%, and 15.84%, 20.43%, and 12.09% for MQ water, lake water, and wastewater, respectively. The decrease in removal percentages could be attributed to the existence of interfering ions, which might mask the active sites on the adsorbent surface. Achieving these results with unmodified activated carbon originating from biowaste is considered plausible. A similar adsorption reduction trend was observed with biochar made of activated carbon from banana peels decorated with nickel sulfide where the target pollutant was ciprofloxacin [85]. The activated carbon by H_3_PO_4_ had dropped after three cycles, however, it remained at 77.10%. The decline in active sites could be attributed to the decrease in adsorption efficiency during three regeneration cycles. Consequently, BPAC demonstrates promise and suitability for the removal of emerging pollutants, indicating its potential for use in environmental remediation. Table 2 presents a comparison of the removal efficiency of contaminants by banana peel residue from several studies with the present study.

## 4. Spent BPAC

It is important to take into account the appropriate disposal of biosorbents in order to prevent any possible harmful effects. The disposal methods for biosorbents include landfilling, incineration, fertilization, regeneration, and reuse [88]. The landfill is an uncomplicated and cost-effective procedure for disposing of waste biosorbents. Before landfilling, the pollutants are desorbed from the biosorbents. Studies have shown that waste adsorbents could be disposed of by burying them in soil or distributing them over land for natural degradation. This strategy might potentially serve as a final means of disposal [89]. Although the cost of the procedure is minimal, the time needed for the natural decomposition of adsorbents is much longer. The incineration process is very efficient because of the abundant presence of lignin and cellulose in the biosorbents. This method aims to decrease both the volume and mass, hence enhancing the total process of recovery [90]. Another way of dealing with the secondary pollution issue of the spent BPAC is to treat the concentrated regeneration streams with effective oxidation methods such as advanced oxidation processes (AOPs). It was found that ultrasound technology as one of the AOPs tested for degrading pharmaceuticals was found to be more efficient for treating concentrated water with pharmaceuticals as opposed to water with low concentrations of these compounds [91]. In the current work, the reuse of the material has been examined and it was successfully used with seventh cycles to remove emerging pollutants in different water matrices.

## 5. Conclusions

This work exhibited the use of banana peel, an agricultural waste, in the development of BPAC, which was revealed to be a very efficient adsorbent for amoxicillin and carbamazepine removal from different waters. After a contact period of 120 min, the adsorption of pollutants from aqueous solutions reached a state of equilibrium. The kinetics investigations suggested that the pseudo-second-order model was more suitable for these contaminants. The analysis of the isotherm demonstrated a strong correlation between the adsorption data and the Langmuir isotherm model. The BPAC had maximal monolayer adsorption capabilities of 393.70 mg/g and 338.98 mg/g for the elimination of amoxicillin and carbamazepine pollutants, respectively. BPAC was easily regenerated and effectively used on seven separate occasions across various water matrices. Based on these data, it can be inferred that BPAC is very beneficial, cost-effective, and efficient in eliminating organic microcontaminants from various aquatic environments. This BPAC could be used as a cost-effective substitute for expensive materials like granular, ion-exchange resins, powdered activated carbons, carbon nanotubes, etc., in the treatment of liquid wastes that include organic micro contaminants. To assess the feasibility of using BPAC as a potential adsorbent for pharmaceutical removal in water treatment facilities, it should be tested with a larger number of contaminants that simulate real-life scenarios.

## Figures and Tables

**Table 1:** Isotherms, kinetics, and thermodynamics equations.

	Equation	Parameter	Reference
Langmuir	$\frac{1}{q_{e}}=\frac{1}{q_{m}}+\frac{1}{q_{m}{\times K}_{L}}\times {{(C}_{e})}^{-1}$	K_L_ is the Langmuir constant (L/mg) and qm is maximum adsorption on BPAC (mg/g)	[39]
Freundlich	${log\;q}_{e}=\frac{1}{n}log\;C_{e}+{log\;K}_{f}$	q_e_ is the pharmaceuticals absorbed per unit adsorbent at equilibrium (mg/g), K_f_ and n are Freundlich adsorption isotherm. constants and C_e_ is equilibrium adsorbate concentration (mg/L)	[40]
Pseudo-first-order (PFO)	$\ln\left(q_{e}-q_{t}\right)=\ln q_{e}-K_{1}t$	K_1_ is PFO constant rate (min^−1^) and q_t_ is pharmaceuticals absorbed at time t (mg/g)	[41]
pseudo-second-order kinetic (PSO)	$\frac{t}{q_{t}}=\frac{1}{K_{2}q_{e}^{2}}+\frac{t}{q_{e}}$	K_2_ is PSO constant rate (g mg^−1^min ^−1^)	[42]
Gibbs free energy (ΔG°), standard entropy (ΔS°) and standard enthalpy (ΔH°)	$∆G{}^{\circ}=-R\times T\times \ln{(K}_{L})$ $K_{L}=\frac{q_{e}}{C_{e}}$ $\ln{(K}_{L})=\frac{-∆H{}^{\circ}}{RT}+\frac{∆S{}^{\circ}}{R}$ ∆G°=ΔH°−T×ΔS°	R is the universal gas constant (8.314 J/mol K), ΔG° is Gibbs free energy, T is temperature (K), ΔS° is standard entropy, ΔH° is standard enthalpy	[43]
Dubinin–Radushkevich (D–R)	$\ln{(q}_{e})=ln\;qd-2Bd\times RT\times In(1+\frac{1}{Ce})$	Bd is the adsorption constant quantifies the average free energy (mol^2^/J^2^), qd is the maximum adsorption capacity (mg/g) T is the temperature (Kelvin) R is the universal gas constant (8.314 J/K/mol)	[44]
Intra-particle diffusion	q_t_ = k_diff_ t^0.5^ + I	K_diff_ is the intra-particle diffusion constant (mg g^−1^ min^−0.5^), I is the IPD model’s boundary layer constant (mg g^−1^)	[45]

**Table 2:** Pollutant removal efficiencies by banana peels from selected studies.

Compound	Matrix	Adsorbent	Removal Efficiency (%)	Reference
Phenol (50 mg/L)	Double-distilled water	Banana peel AC	83	[25]
Phenol (500 mg/L)	Double-distilled water	Banana peel AC	60	[25]
Citric acid	Double-distilled water	Microwave char banana peel	88	[55]
Citric acid	Double-distilled water	Raw banana peel	86	[55]
Rhodamine-B	Double-distilled water	Raw banana peel	81.07	[22]
Atrazine	River and treated waters	Banana peel	>90	[86]
Ametryn	River and treated waters	Banana peel	>90	[86]
Thorium	Wastewater	Banana peel	95.34	[87]
Amoxicillin and carbamazepine	MQ water	Banana peel AC	91.63 and 90.29	Current study
	Lake water		86.63 and 83.29	
	Wastewater		82.34 and 81.51	

## Data Availability

Data are contained within the article and Supplementary Materials.

## Supplementary Materials

The following supporting information can be downloaded at: https://www.mdpi.com/article/10.3390/ma17051032/s1. Table S1: S_BET_ and pore structures of BP samples. Table S2: Langmuir, Freundlich, Pseudo-1st-Order Model and Pseudo-2nd-Order Model parameter. Table S3: Thermodynamic parameters of pollutants adsorption on BPAC.
