# Supplementary material for: A Sustainable Banana Peel Activated Carbon for Removing Pharmaceutical Pollutants from Different Waters: Production, Characterization, and Application
Source: Materials (Basel). 2024 Feb 23;17(5):1032. doi: 10.3390/ma17051032 (PMC10934144; doi:10.3390/ma17051032)
Supplement: Supplementary file 1 [file materials-17-01032-s001.zip › materials-2864652-supplementary.pdf]

A Sustainable Banana Peel Activated Carbon for Removing Pharmaceutical Pollutants from Different Waters: Production, Characterization, and Application [†]

Osamah J. Al-sareji [1,2,*], Ruqayah Ali Grmasha [1,2,3], Mónika Meiczinger [1], Raed A. Al-Juboori [4,5,*], Viola Somogyi [1] and Khalid S. Hashim [6,7,8]

[1] Sustainability Solutions Research Lab, Faculty of Engineering, University of Pannonia, Egyetem str. 10, H-8200 Veszprém, Hungary; ruqayah.grmasha@unswalumni.com (R.A.G.); meiczinger.monika@mk.uni-pannon.hu (M.M.); somogyi.viola@mk.uni-pannon.hu (V.S.)
[2] Environmental Research and Studies Center, University of Babylon, Babylon, Al-Hillah 51001, Iraq
[3] Research Group of Limnology, Center for Natural Science, Faculty of Engineering, University of Pannonia, Egyetem u. 10, H-8200 Veszprém, Hungary
[4] NYUAD Water Research Center, New York University Abu Dhabi, Abu Dhabi 129188, United Arab Emirates
[5] Water and Environmental Engineering Research Group, Department of Built Environment, Aalto University, P.O. Box 15200, FI-00076 Espoo, Finland
[6] School of Civil Engineering and Built Environment, Liverpool John Moores University, Liverpool L3 2ET, UK; k.s.hashim@ljmu.ac.uk
[7] Department of Environmental Engineering, College of Engineering, University of Babylon, Babylon, Al-Hillah 51001, Iraq
[8] Civil Engineering Department, Dijlah University College, Baghdad 00964, Iraq
***** Correspondence: osamah.al-sareji@unswalumni.com (O.J.A.-s.); raed.al-juboori@aalto.fi (R.A.A.-J.)
[†] This research was part of a doctoral thesis for Osamah J. Al-sareji.

Table S1 S_{BET} and pore structures of BP samples

**Samples**	**S_{BET} (m^2/g)**	**V_{tot} (cm^3/g)**	**V_{mic} (cm^3/g)**	**V_{mes}(cm^3/g)**
BP raw	0.650	0.062	0.021	0.041
BP @ 350°C	685.75	0.142	0.044	0.098
BP @ 450°C	911.59	0.180	0.070	0.110
BP @ 550°C	508.62	0.136	0.029	0.107

Table S2 Langmuir, Freundlich, Pseudo-1st-Order Model and Pseudo-2nd-Order Model parameter

**Langmuir**	**qmax (mg/g)**	**K_L**	**R_L**	**R^2**
AMO	393.701	0.037	0.349	0.999
CBZ	338.983	0.058	0.257	0.991
**Freundlich**	1/n	K_f	R^2	
AMO	0.633	21.877	0.982	
CBZ	0.58	28.840	0.777	
**Pseudo-1st-Order Model**	qe (mg g^{-1})	K_1	R^2	
AMO	54.81698	1.46E-05	0.909	
CBZ	73.92123	1.18E-05	0.977	
**Pseudo-2nd-Order Model**	qe (mg/g)	qe^2	K_2	R^2
AMO	1.32E+02	1.73E+04	1.20E-03	0.992
CBZ	1.28E+02	1.64E+04	7.51E-04	0.984

Table S3 Thermodynamic parameters of pollutants adsorption on BPAC

Compound	Temperature (K)	K_l	ΔG°	ΔH°	ΔS°	R^2
AMO	283	5.581	-4.045			0.983
	293	7.799	-5.003			
	303	9.737	-5.733			
	313	11.778	-6.418	18.196	78.841	
CBZ	283	6.243	-4.309			0.976
	293	10.848	-5.807			
	303	15.320	-6.875			
	313	20.407	-7.848	17.116	116.396	
